# Human extracellular matrix (ECM)-like collagen and its bioactivity

**DOI:** 10.1093/rb/rbae008

**Published:** 2024-02-01

**Authors:** Hui Zhou, Wenwei Li, Lixin Pan, Tianci Zhu, Teng Zhou, E Xiao, Qiang Wei

**Affiliations:** State Key Laboratory of Polymer Materials and Engineering, College of Polymer Science and Engineering, Sichuan University, Chengdu 610065, China; Hunan Maybio Bio-Pharmaceutical Co., Ltd, Changsha 410000, China; Hunan Maybio Bio-Pharmaceutical Co., Ltd, Changsha 410000, China; Hunan Maybio Bio-Pharmaceutical Co., Ltd, Changsha 410000, China; Hunan Maybio Bio-Pharmaceutical Co., Ltd, Changsha 410000, China; Hunan Maybio Bio-Pharmaceutical Co., Ltd, Changsha 410000, China; State Key Laboratory of Polymer Materials and Engineering, College of Polymer Science and Engineering, Sichuan University, Chengdu 610065, China; Hunan Maybio Bio-Pharmaceutical Co., Ltd, Changsha 410000, China

**Keywords:** extracellular matrix, native-like collagen, cell-derived matrix, hierarchical structure

## Abstract

Collagen, the most abundant structural protein in the human extracellular matrix (ECM), provides essential support for tissues and guides tissue development. Despite its widespread use in tissue engineering, there remains uncertainty regarding the optimal selection of collagen sources. Animal-derived sources pose challenges such as immunogenicity, while the recombinant system is hindered by diminished bioactivity. In this study, we hypothesized that human ECM-like collagen (hCol) could offer an alternative for tissue engineering. In this study, a facile platform was provided for generating hCol derived from mesenchymal stem cells with a hierarchical structure and biochemical properties resembling native collagen. Our results further demonstrated that hCol could facilitate basal biological behaviors of human adipose-derived stem cells, including viability, proliferation, migration and adipocyte-like phenotype. Additionally, it could promote cutaneous wound closure. Due to its high similarity to native collagen and good bioactivity, hCol holds promise as a prospective candidate for *in vitro* and *in vivo* applications in tissue engineering.

## Graphical abstract

**Figure:**
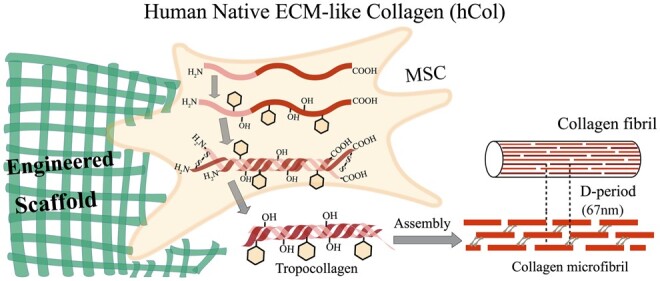

## Introduction

Collagen is the most abundant structural protein of connective tissues including skin, tendon and cartilage in mammals. As a major component of the extracellular matrix (ECM), collagen serves many functions, including regulation of cell adhesion, support for cell migration and guidance of tissue development [1, 2]. Due to its superior biocompatibility and low immunogenicity, collagen has proven safe and effective in a various clinical applications, such as wound healing, skin regeneration, etc. [3–5]. It acts not only as reliable replacement of damaged tissues, but also regulates cell’s biological behaviors and phenotype *in vitro* [6]. The ever-increasing demand for collagen has necessitated the exploration of native or native-like collagen. At present, collagen can be extracted from animal sources such as cows, pigs, bovine or marine life, but the issue of transmissible diseases or lower thermal stability limits the use of collagen from these sources [7, 8]. Additionally, the recombinant collagen can serve as an alternative source to animal collagen; however, it has low structural stability and bioactivity due to a lack of proper post-translational modifications [9, 10]. The production of correctly post-translationally modified recombinant human collagen is very complicated, as the native collagen production requires collagen-specific chaperones, foldases and post-translational modification enzymes [11]. These limitations of native collagen pose obstacles to the widespread application of collagen-based engineered scaffolds *in vitro* and *in vivo.*

Recently, ECM derived from cultured mammalian cells is gaining momentum due to the successful demonstration of glycosylation and key post-translational modifications, which closely mimic the composition and organization of natural collagen [12, 13]. Specifically, the biosynthesis of nature collagen *in vivo*, involving numerous intracellular and extracellular steps, causes it to have a multi-hierarchical fibrous architecture [14]. Similar to biosynthesis *in vivo*, collagen derived by cells can be folded into a triple helix with proper modification and adequate cell ligands to collagen-specific receptors (e.g. α_1_β_1_, α_2_β_1_, α_3_β_1,_ etc.), which can modulate diverse cell functions [15–17]. Besides, collagen derived by cells in the extracellular environment can be further assembled into supramolecular collagen aggregates recognized by an axial periodicity, known as D-period [18]. These advanced structures (triple helix and D-period feature) can be essential for many biological processes or relevant pathological conditions, as they can impact collagen mechanical properties and cell-collagen crosstalk [19, 20]. Although collagen derived from cells has these advantages theoretically, it is important to validate its multi-level structure and its bioactivity.

In contrast to other sources, collagen derived by cells can be customized by selecting the type(s) of cells used to generate the ECM, the culture system (e.g. 2D versus 3D culture), the application of external stimuli to modulate ECM production, etc. Taking advantage of this, there are several methods to generate such matrix proteins like 2D culture systems with protein coating and 3D culture systems (e.g. porous materials [21], microcarrier templating [22] and hydrogel network) [23]. To increase collagen production, we used porous materials with interconnected pores, which are ideal scaffolds for the colonization of seeded cells and matrix accumulation due to large specific surface areas and free space [24, 25]. Polyethersulfone (PES) scaffold was chosen due to its optimal hydrophilicity that benefits protein absorption and further cell attachment. Briefly, the porous PES scaffold was cultured with human mesenchymal stem cells (hMSCs), resulting in the production of matrices composed mainly of types I and III collagen, proteoglycans and glycoproteins. Followed by purification and lyophilization, human ECM-like collagen (hCol) was generated from this collagen-rich ECM containing mainly of types I and III collagen. To validate if native-like properties and hierarchical structures formed in hCol, biochemical and structural characterization of hCol were performed as compared to native animal collagen, including molecular weight, glycosylation, disulfide linkage and protein secondary structure, triple helix and fibril structure. Furthermore, to confirm the bioactivity of hCol, the influences of hCol on stem cell proliferation, migration and differentiation were studied. The novelty of this work is to confirm native-like structures in cell-derived collagen and it is the first time that mesenchymal stem cell (MSC)-derived collagen has been applied *in vitro* and *in vivo.*

## Materials and methods

### Preparation of hCol derived from hMSCs

For the synthesis of a cell scaffold, PES was used as a cell culture platform because of its proper hydrophobicity to enhance protein adhesion for cell growth. Briefly, an 8 wt % PES solution was extruded into a water bath by liquid-liquid phase separation and dissolved in *N*,*N*-dimethylacetamide under ultrasound for 15 s. Water was then added to terminate the dissolution, resulting in the synthesis of PES scaffolds. Subsequently, the scaffolds were thoroughly washed in boiling water to remove the solvent, followed by steam, alcohol and UV sterilization before cell culture. hMSCs were then seeded at a density of 1 × 10^5^/cm^2^ in the scaffolds with a customized medium [26]. After a 14-day culture, the scaffolds were decellularized and purified to collect collagen-rich ECM following a protocol that was similar to that previously described [8, 27]. Briefly, the scaffolds were treated with a precooled 0.25% trypsin-EDTA solution for 20 min, followed by another 20-min treatment with the addition of 3% Triton X-100. Afterward, the scaffolds were washed with deionized water and freeze-dried. To remove PES materials, the scaffolds were dissolved in DMSO followed by a 10-min centrifugation step at 8000 rpm. The ECM was then collected by washing the precipitate with DMSO and deionized water, respectively, three times each. Subsequently, the ECM was treated with 0.5 M acetic acid for 24 h at 4°C, then centrifuged and pepsin (1 mg/mg protein) was added overnight. Afterward, the supernatant was salted-out with NaCl for 12 h and dialyzed against 0.1 M acetic acid with a molecular weight of 7 kDa before being lyophilized and stored at −20°C until further use.

To monitor *in vitro* expansion of stem cells, the hMSC cell line was transfected with retroviral vectors (GeneChem, NM_009354) containing the human telomerase reverse transcriptase (hTERT) gene according to the manufacturer's protocol. The immunofluorescence of transduced cells (hTERT-hMSCs) using rabbit anti-TERT antibodies (Abcam, Ab191523) was captured under fluorescence microscopy (Leica DMi8, Leica Microsystems). The hTERT-hMSCs were cultured in standard Dulbecco’s modified eagle’s medium (DMEM; Gibco, 21885-025) supplemented with 10% fetal bovine serum (FBS; Gibco, 10099141C) and 1% penicillin/streptomycin (PS; Gibco, 15140122) at 37°C with 5% CO_2_.

### Biochemical characterization of hCol

#### SDS-PAGE

Lyophilized samples of bovine type I collagen (bCol I) and hCol were dissolved at a concentration of 0.2 mg/ml in 3% acetic acid at 4°C for 24 h. The dissolved samples were mixed at a 4:1 ratio (v/v) with the sample buffer (0.5 M Tris–HCl, pH = 6.8, containing 20% SDS (10% w/v), 10% beta-mercaptoethanol, 0.5% bromophenol blue and 20% glycerol) and boiled for 5 min. Then the protein samples (20 μg/well) were loaded on 12.0% polyacrylamide gel including a 4% stacking gel, and subjected to electrophoresis at a constant voltage of 80 V for 15 min and then at 120 V for 15 min. The gels were further stained with Goomassie G-250 to capture images.

#### Mass spectrometry (LC-MS/MS)

The lyophilized samples (hCol and bCol I) were digested with enzymes (Promega), including trypsin, chymotrypsin & Glu-C, chymotrypsin, trypsin & Glu-C and elastase for glycan analysis, while the samples were digested with trypsin and Glu-C for disulfide bond analysis. Then, the digested proteins (0.2 μg each) were analyzed on an Easy-nLC 1200 with a nanospray source connected to a Q Exactive Hybrid Quadrupole-Orbitrap mass spectrometer (Thermo Fisher Scientific, Waltham, MA). The top 10 most abundant precursor ions were selected from a 300-*m/z* to 1800-*m/z* full scan for collisional activation dissociation (HCD). The raw files were analyzed and searched against the target protein database based on the species of the samples using PLINK software version 2.3.5 (Christopher Chang/Grail Inc) [28]. The maximum missed cleavages were set to 3; the precursor ion mass tolerance was set to 20 ppm, and the tolerance was set to 0.02 Da.

### Structural characterization of hCol

#### FTIR

The lyophilized bCol I and hCol were examined by Fourier transform infrared spectrometry (FTIR, Nicolet 560, America). Both the background and samples were scanned with 32 accumulations in the wavenumber range of 4000–500 cm^−1^. The spectra were processed using automatic baseline calibration and 11-point Savitzky–Golay smoothing with the order of 3 for further analysis.

#### Raman spectroscopy

Raman spectroscopy was performed on the sample in a labRam HR evolution microscope (HORIBA France SAS, Palaiseau, France) with a 532-nm continuous wave solid-state laser and a 50× air lens (NA = 0.5). The spectra were collected from 600 to 1800 cm^−1^ with 12 accumulations and an acquisition time of 10 s each. The raw spectra were processed by subtracting background fluorescence using a fifth-order polynomial fit and smoothed by Savitzky–Golay filters with an order of 3 and a window size of 11 in Labspec 6.0 software (Horiba, France) as previously described [29]. The spectra were then processed with standard normal variate normalization in Matlab^®^. The processed amide I band in the range of 1600–1700 cm^−1^ was decomposed in multiple sub-bands based on the Levenberg–Marquardt algorithm in OriginPro 2021 (OriginLab Corporation, Northampton, MA, USA). All the secondary structural content was estimated by dividing the areas under each sub-band by the whole area of the amide I band and reported as a percentage.

#### Circular dichorism spectroscopy

Circular dichorism (CD) spectra of hCol and bCol I (0.25 mg/ml in pH 12 NaOH) were obtained using a Chirascan V100 Spectrometer (Applied Photophysics Ltd, UK) using 0.5-mm quartz cuvettes. The spectrum (190–260 nm) for the NaOH solution (pH 12) was subtracted from each sample spectrum and then smoothed using Savitsky–Golay with a polynomial order of 2 and a window size of 3. The molar ellipticity [Θ] was calculated by the following equation:

(1) $$\left[\Theta \right]=(\Theta \times 100\times M_{w})/(C\times l)$$

where Θ was ellipticity obtained from CD spectrometer, Mw is the molecular weight of amino acid residue, *C* is the concentration of sample and *l* is the width of cuvette. The thermal curves of triple helix were monitored by the molar ellipticity at 222 nm with increasing the temperature from 22°C to 55°C at a rate of 1°C/min.

#### Electron microscopy

The scanning electron microscopy (SEM) was performed using an Apreo S HiVoc (Thermo Fisher Scientific, FEI) operated at 5 kV. All SEM samples were observed with gold coating with a layer of about 5 nm. Droplets (10 μl, 0.004 mg/ml) of the protein solution in NaOH (pH 9) are placed on ultrathin carbon film on copper grids (Ted Pella, Inc., USA). The transmission electron microscopy (TEM), aberration-corrected high-angle annular dark-field scanning TEM (AC HAADF-STEM) and energy dispersive spectroscopy mapping were achieved on Bruker Nano GmbH Quantax (ThermoFisher Scientific, USA) using an electron acceleration energy of 200 kV with a cold field emission electron source.

### The effect of hCol on cell behaviors and phenotype

#### Cell proliferation

To study the effect of hCol on cell proliferation, the cell viability of hASCs was measured using CCK‐8. The hASCs were planted into 96‐well plates (2000 cells per well) and CCK‐8 solution (10 µl/well) was added to measure cell viability. The optical density (OD) at 450 nm wavelength was measured using the Multiskan FC (Thermo Fisher Scientific, Inc, Waltham, MA, USA). The cell growth was quantified by counting number of cells per ml in cell suspension using a hemocytometer at different hours after cell seeding and bright filed images were taken before counting.

#### Cell migration

A scratch test was used to determine the effect of hCol on the migration ability of human adipose stem cells (hASCs). Briefly, hASCs were cultured on 6-well plates (5 × 10^4^/ml per well) until reaching 90% confluence using complete growth medium (10% FBS/1%PS/DMEM) and then washed with PBS. Cell scratches were made with a 200-µl pipette tip and the bright field images were taken at 0, 24 and 48 h after the scratch. For comparison of the effect of different collagen groups on cell migration, hASCs were cultured respectively in a medium supplemented with 10 μg/ml hCol, a medium with 10 μg/ml bCol I and a complete growth medium alone (blank control). The migration rate was calculated using the wound area closure equation described previously [30]. To explore if the structure of hCol plays a role in cell migration, hCol was exposed to the following processes and then used in cell migration in the same way: alkaline, steam sterilization, vapor hydrogen peroxide (VHP) and radiation treatment, respectively. For alkali treatment, hCol was treated with a solution of 3.0% NaOH (w/v), 1.9% monomethylamine (v/v) at 20°C. For steam sterilization, hCol was autoclaved at 140 kN/m^2^ steam pressure at 126°C for 11 min. For VHP, hCol was kept in a peroxide solution at room temperature for 30 min. For radiation, hCol was exposed to a radioactive cobalt-60 source at ambient temperature until a minimum dose of 25 kGy was achieved.

#### Cell differentiation

To differentiate cells, hASCs were seeded at a density of 1 × 10^5^ per well in a 6-well plate with adipogenic induction medium (AIM) that contains 1 μM dexamethasone, 0.5 mM 3-isobutyl-methylxanthine, 10 μg/ml insulin and 0.2 mM indomethacin (Sigma-Aldrich, St Louis, MO, USA). For blank control, hASCs were cultured in a complete growth medium (DMEM/10%FBS/1%PS). To study the effect of hCol on cell differentiation, hASCs were cultured on a 6-well plate coated with 1 mg/ml hCol in the AIM medium and compared with those in the AIM medium and complete growth medium alone, respectively. After 14-day culturing, total RNA was extracted to analyze differentiation markers, including peroxisome proliferator-activated receptor (PPAR2γ) and key enzyme gene lipoprotein lipase (LPL). After 21 days of differentiation, cells were fixed with 75% ethanol and stained with Oli Red O solution (Cyagen Biosciences Inc., OILR-10001) for 30 min. Bright-field images were acquired on a Leica DMi8 Microscope. For gene expression, each RNA sample (1 μg) was used to synthesize cDNA with a PrimeScript^®^ RT Reagent Kit (Takara, Japan) following the manufacturer’s recommendations. The PPARγ2 gene was amplified and quantified using the forward primer 5′-GGG TGA AAC TCT GGG AGA TTC TC-3′; reverse primer 5′-GAT GCC ATT CTG GCC CAC-3′ and the LPL gene was amplified and quantified using forward primer 5′-ATTCTCCCTCCGCAAACCC-3′ and reverse primer 5′-GGAGGTGCTGTTGAAGGTG-s′. At reverse transcription reaction, cDNA (SYBR Green I, Invitrogen, Carlsbad, CA, USA) was used as the template for real-time quantitative PCR for LPL and PPARγ2 while glyceraldehyde-3-phosphate dehydrogenase (GAPDH) was utilized as an internal control. The typical cycling conditions consisted of an initial denaturation step at 94°C for 4 min, 35 cycles of amplification at 94°C for 20 s, 60°C for 30 s and 72°C for 30 s. The relative expression of the gene was measured as the relative expression of mRNA in the indicated groups to control (normalized to the corresponding GAPDH values).

### The effect of hCol on wound closure in mouse tail skin

All procedures involving the use of animals in this study were prospectively reviewed and approved by the Institutional Animal Care and Use Committee. This study was conducted in adherence to the guidelines and was approved by the ethical committee (no. 2021911 A) of West China Hospital, Sichuan University. During the experiment, the animals had free access to water and food. Here, five 6- to 8-week-old male SPF BALB/c mice with a body weight of 18–20 g were used for this study. The full-thickness wounds corresponding to the template area (10 × 5 mm) were created to the dorsal surface of the mice’s tail by sterile gauge scalpels (Becton Dickinson Co., Hancock, NY, USA). For the experimental group, hCol (10 μg/ml) was applied to the wounded areas twice daily while the commercial recombinant collagen (10 μg/ml, Shanxi Jinbo Bio-Pharmaceutical) was applied as control in the same way. Photographs of the wounds from a fixed distance at regular time intervals were taken. After inducing anesthesia with an excessive dose of isoflurane, the mice were euthanized through cervical dislocation. The wound sites after day 14 were paraffin-sectioned, hematoxylin and eosin (H&E) staining was performed and the images were acquired on a Leica DMi8.

### Statistical analysis

Statistical analyses were performed using the software JMP Pro 12 (SAS company, Car, NC, USA). An unequal variance *t*-test (two-sample *t*-test) would be used for comparing proliferation as well as adipogenesis (between AIM group and AIM + hCol group). A pooled two-sample student *t*-test was used for comparing wound closure areas. *Post hoc* Dunnett’s test was used to determine the significance of hTERT intensity among different culturing days compared to control, cell migration treatments compared to control (no treatment) as well as gene expression among different medium as compared to control medium. *P* ≤ 0.05 was considered statistically significant.

## Results

### Preparation of human ECM-like collagen

**Figure 1.**
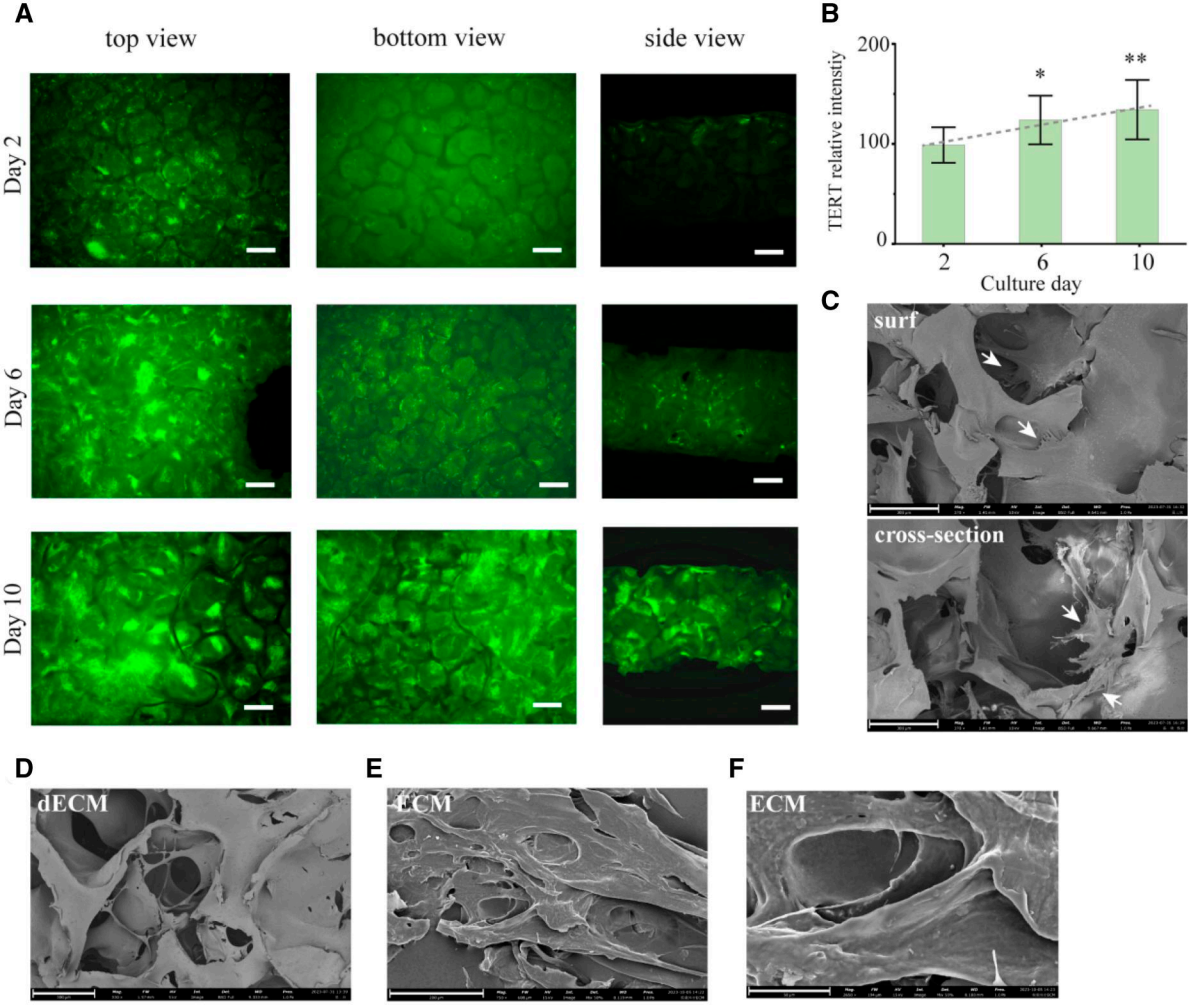
hTERT-hMSC growth and morphology of PES scaffold and ECM. (**A**) Representative confocal 3D scanning images of the apical surface (top view), the basal surface (bottom view) and side view of PES scaffold subjected to staining for hTERT (green) after culturing for 2, 6 and 10 days, scale bar= 500 μm. Statistical difference: *P < 0.05 and **P < 0.01 compared with the day 2 (control) when cells start to express hTERT. (**B**) The relative TERT fluorescent intensity change in PES scaffolds with different culturing days. Data shown are the means ± SD for three independent experiments. SEM images of (**C**) the surface and the cross-section of PES scaffold (day 10), (**D**) the scaffold surface after decellularization (day 14), (**E**) ECM after removing PES materials and (**F**) ECM at higher magnification. The scale bars: 300 μm (C and D), 200 μm (E) and 100 μm (F).

To confirm if the PES culture platform is suitable for producing cell-derived matrix, the growth of hTERT-hMSCs was monitored. As shown in Figure 1A and B, the continuous increase of TERT expression with culturing days indicated the well growth of hMSC and ensures a critical mass of hMSCs without influence of transcription. The SEM images showed pores in PES scaffolds, each about a few hundred micrometers wide, where hMSCs can grow (Figure 1C). After 10 days of cell seeding, cells were well spread over the surface and cross-section of porous PES scaffold with cell membrane extensions (filopodia and lamellipodia). Cells merged completely and intercellular connections were barely visible, suggesting that PES scaffolds had good biocompatibility for hMSC attachment. The decellularization before collecting ECM got rid of cellular components (Figure 1D) and the PES template was further removed (Figure 1E and F) without minimal damage to the scaffold structure.

### Biochemical characterization of human ECM-like collagen

**Figure 2.**
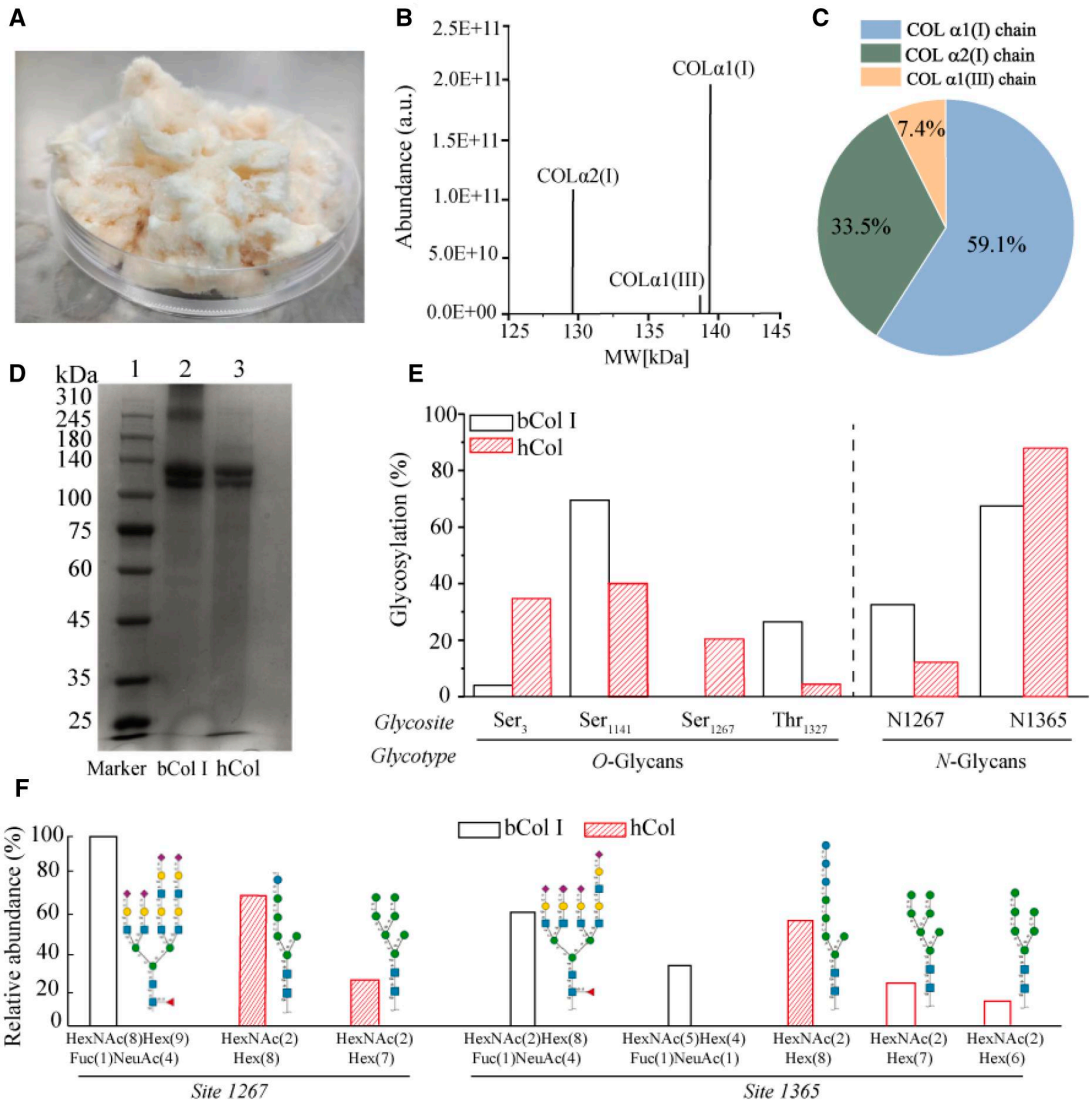
Biochemical characterization of hCol as compared to native bCol I. (**A**) The photo of purified massive hCol. (**B**) Mass spectrum of hCol as a function of molecular weight (MW). (**C**) Most abundance of collagen chain in hCol as detected by mass proteomics. (**D**) Protein gel electrophoresis of the collected hCol from the PES scaffolds. Lane 1 is molecular weight ladder and lanes 2 and 3 are from the samples of bCol and hCol. (**E**) The percentage of O-glycosites and N-glycosites in bCol I and hCol, respectively. (**F**) The relative abundance of N-glycan composition for N1267 and N1365 between bCol I and hCol (upper pannel: bCol I; lower pannel: hCol).

The collected ECM was purified and lyophilized to obtain massive hCol as seen in Figure 2A. The mass proteomics analysis demonstrated that the relative staining intensity of α1 band was higher than that of α2 band in both groups. Considering the native type I collagen mostly occurs as a heterotrimer with two identical α1-chains and one α2-chain [31], the mass analysis implied that hCol was mainly composed of type I collagen (Figure 2B and C). This was in agreement with SDS-PAGE data, where the percentage of α1-chain is about 1.8 times of α2-chain as seen in Figure 2D. The electrophoretic protein pattern showed hCol was composed of α1 chains (132 kDa) and α2 chains (122 kDa), which was similar to native bovine collagen (bCol I) consisting of α1 (132 kDa) and α2 chains (123 kDa), as seen in Figure 2D. Furthermore, the quantitative glycoproteomic data were then analyzed to investigate whether hCol showed distinct glycopeptide features as compared with bCol I. As shown in Figure 2E, collagen chains were glycosylated at Ser_3_, Ser_1141_ and Thr_1327_ sites in both bCol and hCol, though the glycosylation at Ser_1267_ was present in hCol while absent in bCol I. Among all O-glycan sites, the modification at Ser_1141_ appears to be most abundant for both bCol I and hCol. For N-glycosides, N1267 and N1365 were observed to be modified and N1365 is predominantly present in both groups (Figure 2F). These observations collectively indicated a very similar pattern of glycosylation on collagen chains for both groups. However, N-glycan structures between bCol I and hCol were different to some degree. All N-glycans between the two shared a common core structure including the first two N-acetylhexosamine (HexNAc) residues and the first three hexose (Hex) residues. Nevertheless, N-glycans of hCol exist as high-mannose type consisting of only HexNAc and Hex residues, while N-glycans of bCol I exist as complex type with additional sugars such as fucose (Fuc) and sialic acid (NeuNAc).

Before assembling into suprastructures, the inter-chain disulfide bonds between the C-propeptides of collagen are formed as in animals, which requires chain selection and collagen-specific molecular chaperones [32]. Therefore, the confirmation of the presence and location of the disulfide bond was obtained from the MS/MS spectrum (Supplementary Figure S1). A series of b/y-type ions from backbone cleavage between collagen α chains were both generated in bCol I and hCol (see Table 1). For α1 to α2 chain, ions with *m/z* of 74.06, 147.11 and 175.12 were detected for hCol while no valuable ion fragments were observed for bCol I. Nevertheless, 518.28 and 781.37 ions for bCol I as well as 102.06, 166.09, 215.10, 245.11, 385.21, 690.33, 713.89 and 718.84 ions for hCol were all generated by the cleavage of disulfide bonds from the peptides that had same amino acid sequence (TCIRAQPE-VDIGPVCF). This confirmed the disulfide bond formed at the same site for two groups. Besides, the fragment ions from bCol I (*m/z*: 205.08, 212.10, 283.14, 662.34 and 725.34) and the ions from hCol (*m/z*: 175.12, 356.20 and 382.17) were both related to the same amino acid sequence (NPARTCR-FCHPE) with cysteines (Cys) connected vis S–S bond between α2 chain and α3 chain. Moreover, two groups also showed the same amino acid sequence (TCISANPLNVPR-DGCTKHTGE) within α3 chains, with Cys cleaved as indicated by *m/z* 387.20 for hCol as well as 74.06, 102.06, 159.08 and 175.12 ions for bCol I. All disulfide linkages were formed at the same sites between α chains in hCol as in native bCol I, though the lengths of fragments were different.

**Table 1.** Ions observed in the spectra of disulfide peptide for bCol I and hCol^a^

Group	Peptide sequence	Fragment ions	Assignment of fragment ions	Charge z	*m/z*
bCol I	TCIRAQPE-VDIGPVCF	AKNW	y4 (CoI α2 chain)	1+	518.28
		DIGGADQEFFVDIGP	b15 (CoI α1 chain)	2+	781.37
	NPARTCRDLR-FCHPE	GE	β2 (CoI α3 chain)	1+	205.08
		NP	b2 (CoI α2 chain)	1+	212.10
		NPA	b3 (CoI α2 chain)	1+	283.14
		ELKSGE	y6 (CoI α3 chain)	1+	662.34
		CFC	b2 (CoI α3 chain)	2+	725.34
	CISANPLNVPR-DGCTKHTGE	GNSK	b4 (CoI α3 chain)	1+	387.20
hCol	DLKMCHSDWK-NPARTCRDLR	K	y1 (CoI α1 chain)	2+	74.06
		K	y1 (CoI α1 chain)	1+	147.11
		R	y1 (CoI α2 chain)	1+	175.12
	TCIRAQPE-VDIGPVCF	T	b1 (CoI α2 chain)	1+	102.06
		F	y1 (CoI α2 chain)	2+	166.09
		VD	b2 (CoI α2 chain)	1+	215.20
		PE	y2 (CoI α2 chain)	1+	245.11
		VDIG	b4 (CoI α2 chain)	1+	385.21
		PVCF	y4 (CoI α2 chain)	2+	690.33
		IRAQPE	y6 (CoI α2 chain)	1+	713.39
		GPVCF	y5 (CoI α2 chain)	2+	718.84
	NPARTCR-FCHPE	R	y1 (CoI α2 chain)	1+	175.12
		GSRKNPA	b7 (CoI α2 chain)	2+	356.20
		HPE	y3 (CoI α3 chain)	1+	382.17
	CISANPLNVPR-DGCTKHTGE	K	y1 (CoI α3 chain)	2+	74.06
		T	b1 (CoI α3 chain)	1+	102.06
		TG	b2 (CoI α3 chain)	1+	159.08
		R	y1 (CoI α3 chain)	1+	175.12
		DGCT	b4 (CoI α3 chain)	2+	973.45

a Peptide sequence is the same sequence shared between hCol and bCol I.

### Structural characterization of humanized ECM

#### Molecular structure of hCol

**Figure 3.**
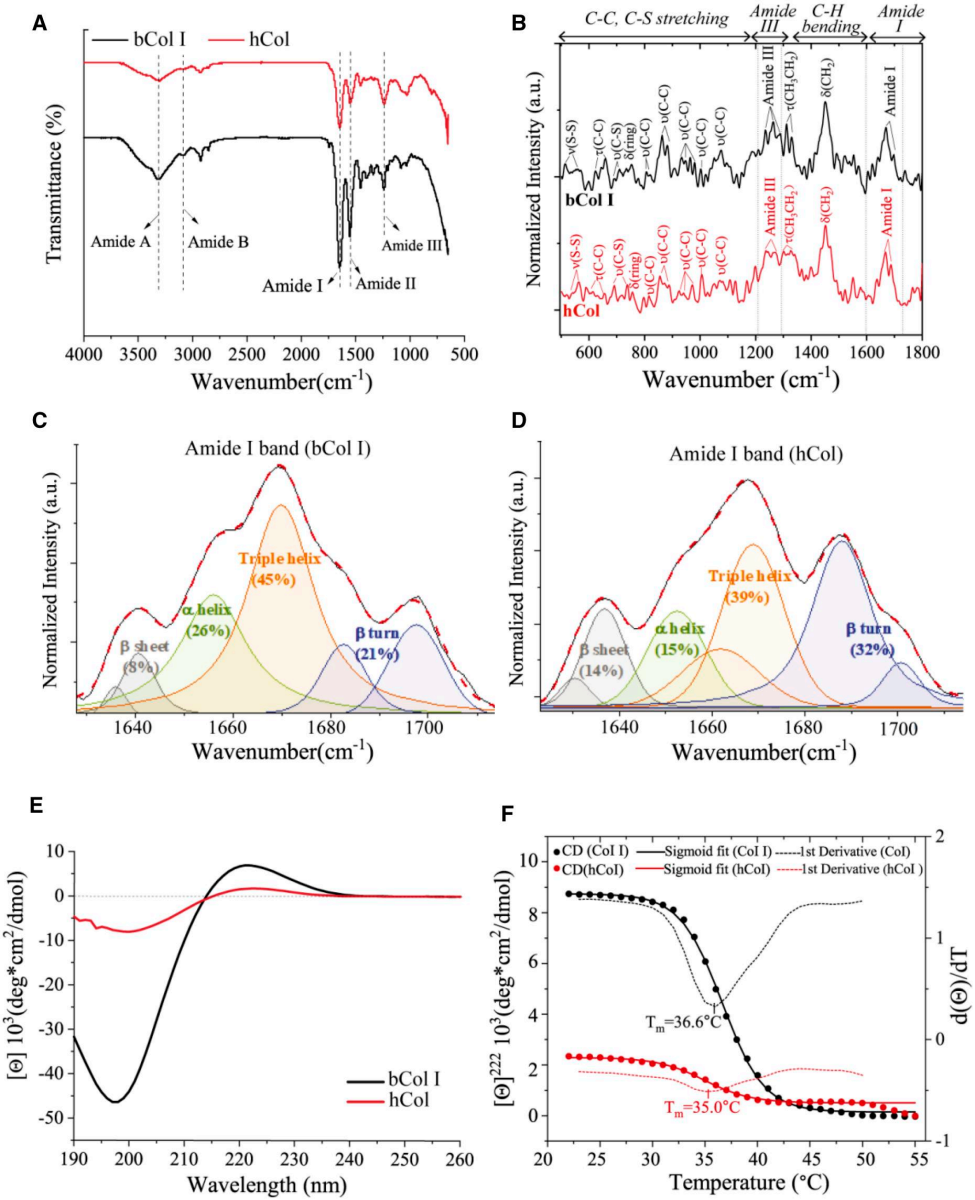
The molecular structure analysis of hCol and bCol I. (**A**) FTIR spectra and (**B**) Raman spectra of bCol I and hCol. The decomposition of amide I Raman band for (**C**) bCol I and (**D**) hCol. (**E**) The CD spectra of bCol I and hCol at room temperature 22˚C. (**F**). mean residue molar ellipticity at 222 nm [Θ], as a function of temperature for bCol I (black dot) and hCol (red dot) which is fitted with the sigmoid equation. The first derivatives from sigmoid fits were shown dashed lines.

As seen in Figure 3A and Supplementary Table S1, FTIR spectra of hCol and bCol I showed the same wavenumber of amide A and amide I, while exhibiting redshifts in amide B, amide II and amide III (predominantly N-H bending vibrations). These redshifts indicated a higher proportion of hydrogen bonds for NH groups, which hold the collagen strands together in hCol. The Amide III peak to υ (CH_2_) peak (at 1450 cm^−1^) ratio was usually used to determine the degree of unique secondary structure-triple helix, which was about 1.0 for native collagen [33]. Here, this ratio in hCol (0.98) was slightly lower than bCol I (1.0), suggesting a lower degree of triple helix structures in hCol as compared to native collagen. Furthermore, the secondary structure in detail was investigated using Raman spectroscopy, and the characteristic peak assignments of bCol I and hCol in the region 600–1800 cm^−1^ were listed and explained in Supplementary Table S2. The spectra region of 600–1200 cm^−1^ in both spectra was dominated by S–S, C–S and C–C stretching of amino acids such as hydroxyproline, proline, tyrosine, cystine, tryptophan and phenylalanine (Figure 3B). In particular, the presence of hydroxyproline peak at 816 cm^−1^ for hCol supported the hydroxylation of proline residues in collagen alpha chains, which can be required for the stability of the collagenous triple helix at physiological temperatures. For amide I band, it has been proven to be the most sensitive and popular band to study protein secondary structure, with each type of secondary structure correlated to a slightly different C = O stretching frequency due to its unique molecular geometry [34]. The amide I band was decomposed to show different secondary structures and amide I of hCol was dominated by the triple helix, with its abundance lower than that in bCol I (Figure 3C and D), which was consistent with findings from FTIR investigations. After determining the presence and abundance of triple helix structure, the thermal stability of triple helix structure was characterized using CD. Both bCol I and hCol showed a positive peak around 222 nm (Figure 3E), indicating hCol had a similar triple helix structure as native bCol I. The melting behavior of the triple helix was monitored by the change of ellipticity at 222 nm and transition mid-temperature (*T*_m_) due to denaturation of the triple helix was obtained from the first derivatives of sigmoidal fitting of the melting curves. As shown in Figure 3F, *T*_m_ of hCol was 35°C and was slightly lower than that of bCol I (*T*_m_ = 36.6°C), indicating a slightly lower degree of conformational stability in hCol. Nevertheless, the degradation temperature for both bCol I and hCol was above 40°C, suggesting stability of the triple helix structure of hCol under normal physiological conditions.

#### Morphology analysis of fibrillar structure in hCol

**Figure 4.**
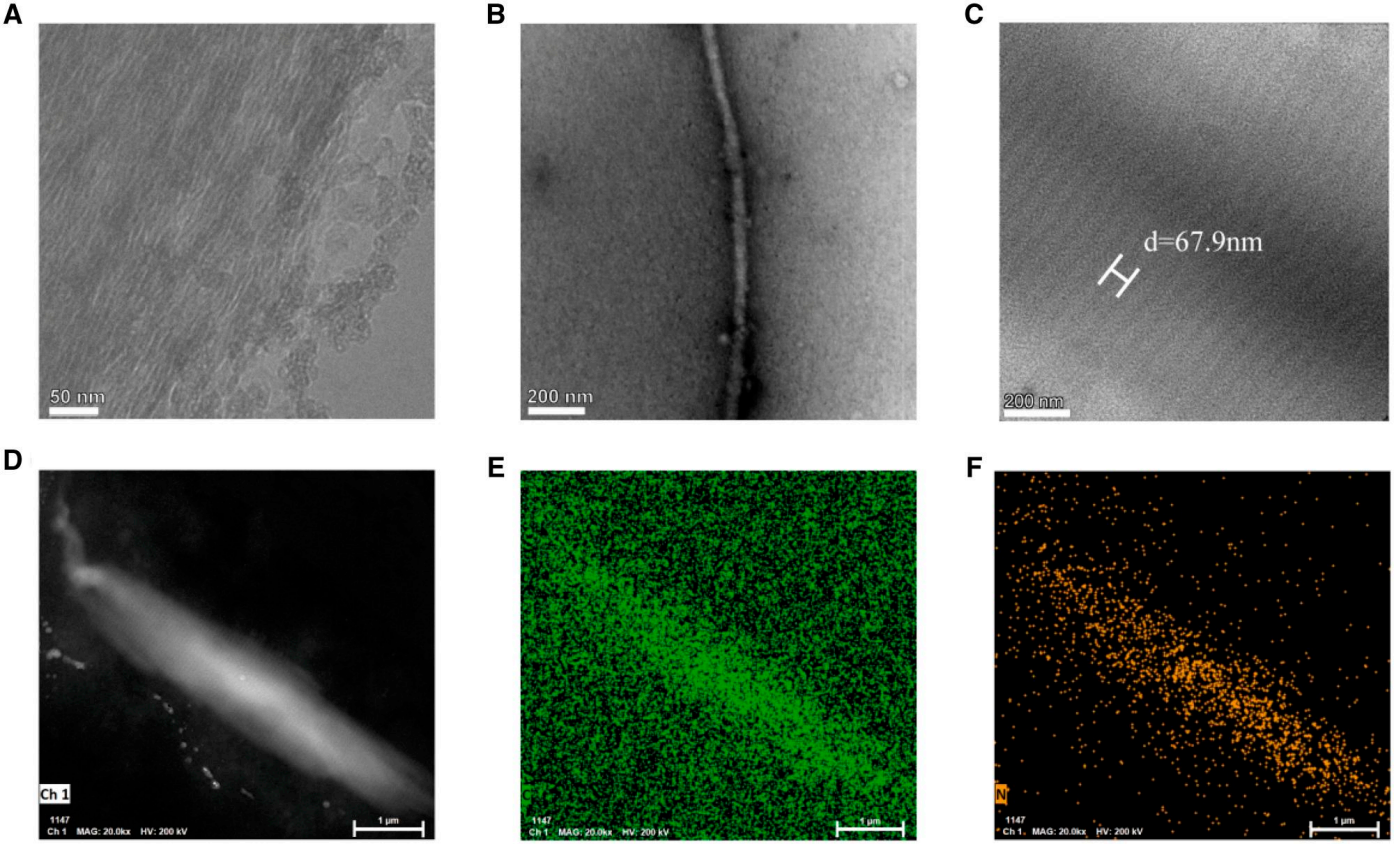
The morphology analysis of fibril structure in hCol. TEM images of (**A**) a cluster of collagen fibrils, (**B**) individual fibril and (**C**) D-periodicity within fibrils. (**D**) The HAADF-STEM image and EDS mapping of the collagen fibril for (**E**) carbon element and (**F**) nitrogen element.

Fibrillar collagens, including type I and type III are higher order, supramolecular assemblies of triple helices to form fibril structures [35]. As shown in Figure 4A, a dense crust of highly aligned fibrils was formed within fiber structures in hCol. As to well-distributed collagen fibrils in solution, a fibril with a diameter of around 30 nm was observed (Figure 4B), which fell within the ranges of collagen fibril diameters reported in the literature (20–500 nm) [36]. Besides, alternate light/dark-band patterns were observed in the fibril of larger diameter (Figure 4C), which was associated with the regular arrangement of the collagen monomers (termed D-period). Here, the measured D-period was around 68 nm, consistent with literature values for fibrillar collagen [19, 37]. The HAADF-STEM image of the fibril was applied with EDS analysis and strong peaks of carbon and nitrogen elements were observed in hCol (Figure 4D–F). All the above results demonstrated native-like higher-order structures formed in hCol.

### Influence of hCol on cell behaviors and cell phenotype

Collagen plays an important role in regulating cell behaviors and phenotype, not only due to abundant cell recognition sites but also its unique structure form [38–40].

Cellular interactions with engineered collagen are largely affected by how closely the collagen is able to recapitulate the structure of native collagen on different length scales [41]. Multilevel hierarchical structures were observed in hCol, which might affect a series of biological cell behaviors, including proliferation, migration, differentiation and more.

**Figure 5.**
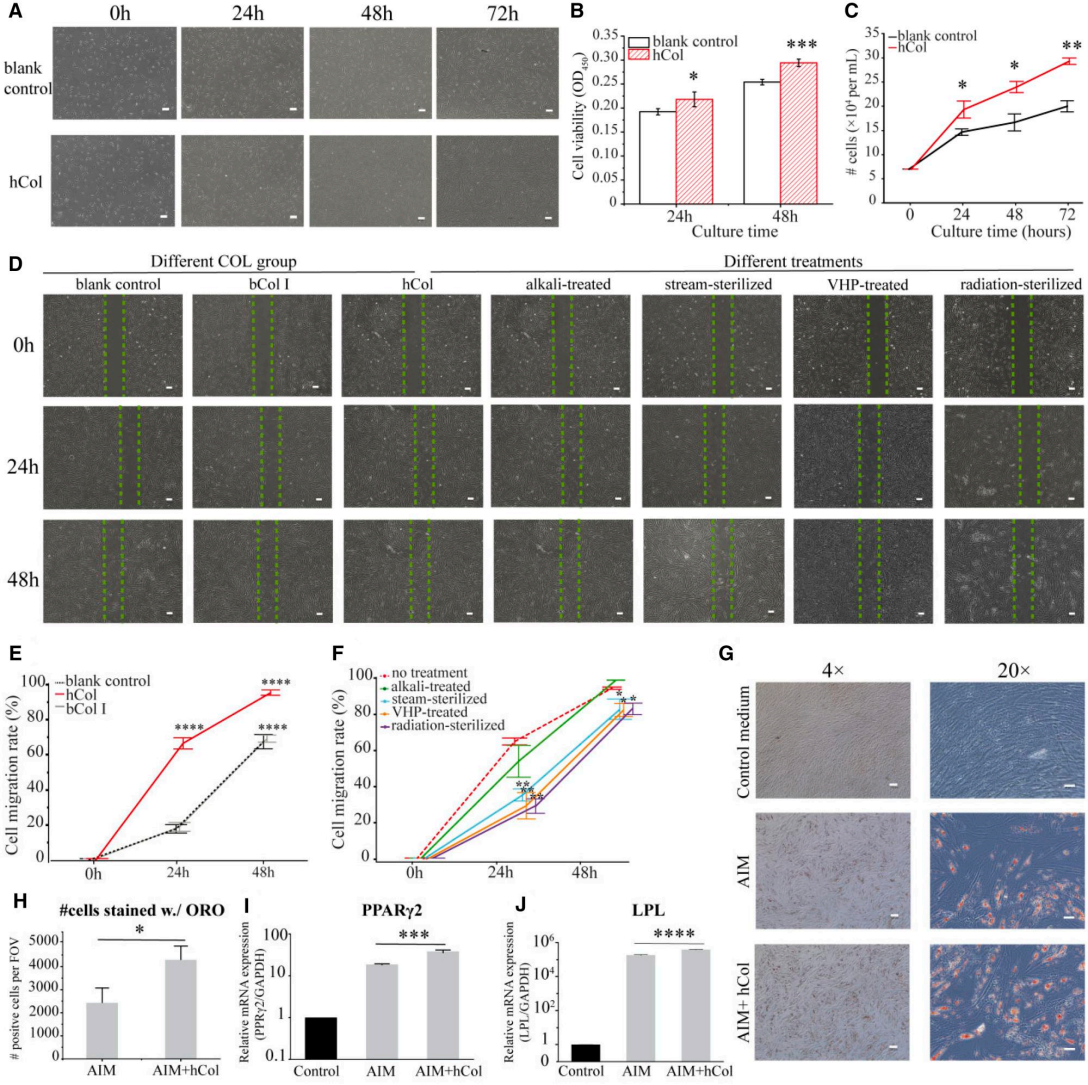
hCol Promotes cell proliferation, cell migration and adiopenesis of hASC. (**A**) Representative images show cell confluency at different times (blank control: complete growth medium alone; AIM: adipogenic induction medium alone; AIM+ hCol: seeded on hCol-coated surface in AIM medium), scale bar =200 μm. (**B**) CCK-8 assay: OD450 absorbance in hASCs cells (mean ± SD, *n* = 4). (**C**) Cell growth curve determined by cell count after 0, 24, 48 and 72 h of hASC cells exposure to hCol media (mean ± SD, *n* = 3). (**D**) representative images of cell migration in different collagen (COL) groups and different treatment groups, scale bar = 50 μm. (**E**) The cell migration rate of different COL groups (means ± SD, *n* = 3). (**F**) The migration rate of cells exposure to hCol with different treatments (means ± SD, *n* = 2). (**G**) The representative images of Oil Red O staining of hASC (4×: scale bar = 200 μm; 20×: scale bar = 50 μm). (**H**) The quantified result for Oil Red O-positive hASCs cultured in control growth media, AIM and AIM + hCol. The relative expression of (**I**) LPL and (**J**) PPARγ2 gene normalized to GAPDH mRNA expression. (mean ± SD, *n* = 3). Statistical difference: *P < 0.05, **P < 0.01, *** *P* < 0.001 and **** *P* < 0.0001 as compared with blank control at each time point.

First, the influence of hCol on cell viability and cell proliferation was evaluated using a CCK-8 assay and brightfield cell counting (Figure 5A), respectively. As seen in Figure 5B, the cell viability of hASCs indicated by absorbance at OD 450 nm increased with the addition of hCol in the cell medium (10 μg/ml) compared to the blank control (cell medium only) after 24 and 48 h of seeding. The number of hASCs increased with culturing time and the significant difference in the cell number in hCol was greater compared to blank control (Figure 5C). Besides, the effect of hCol on cell migration was detected by scrape motility assays. As seen in Figure 5D and E, the scratching test showed that the hASC migration rate was greater in hCol as compared to bCol I and blank control. The collagen structure has been experimentally shown to influence cell migration, which might get side-lined without its triple helix and fibrillar structure [42, 43]. Hereby, to test if this is related to the native-like structure of collagen, the cell migration was investigated for hCol with different treatments. Except for alkali-treated hCol, the migration rate was lower in all treatment groups as compared to no treatment (Figure 5F). Furthermore, to analyze the effect of hCol on cell phenotype, Oil Red O staining was used to indicate adipogenic differentiation. The hASCs cultured in the control expansion medium did not undergo adipogenesis, which did not stain positive with Oil Red O (Figure 5G). The hASCs cultured in the AIM differentiation medium were differentiated, showing positive Oil Red O staining. The cells seeded on the hCol-coated surface in the AIM medium were differentiated and more and bigger lipid droplets were detected as compared to the AIM group. AIM + hCol group yielded higher percentages of lipid-positive cells, with significant differences as compared to AIM (Figure 5H). When hASCs differentiate into adipocytes, several differentiation-linked genes are activated, among which LPL and peroxisome proliferator-activated receptor γ2 (PPARγ2) are important genes induced in the adipogenic differentiation process. The differentiation in the AIM + hCol group showed expression of LPL by 2-fold increased and expression of PPARγ2 by 2-fold increased as compared to AIM group (Figure 5I and J).

### The effect of hCol on wound healing

**Figure 6.**
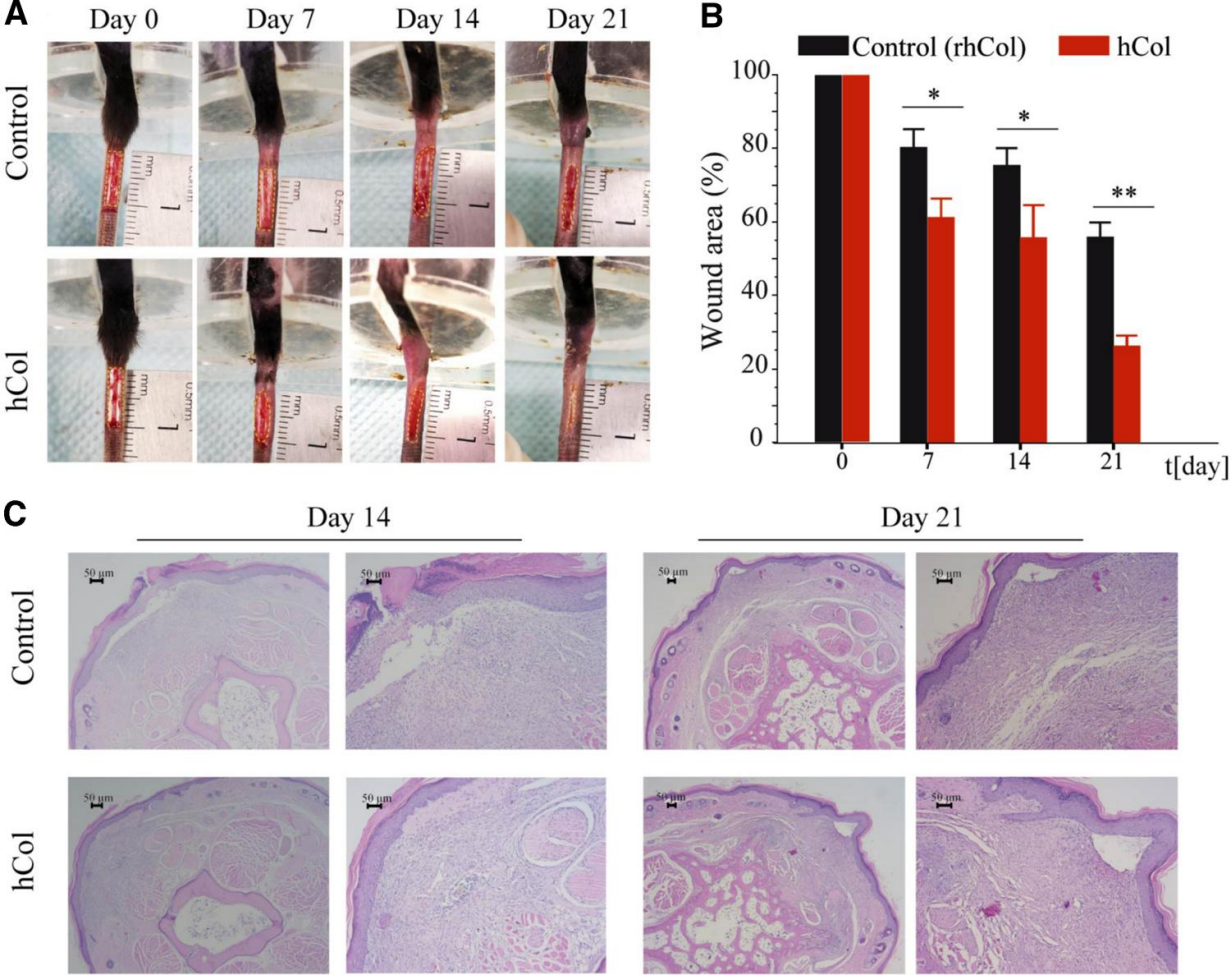
The effect of hCol on wound healing in mouse tail skin. (**A**) Representative photographs of mouse tail skin days 0, 7, 14 and 21 after wounding (control: commercial collagen). (**B**) The quantified wound closure area normalized to day 0 post-operation. (**C**) HE staining of cross-section of mouse tail 14 days and 21 days after operation. All bars shown in (B) represents mean ± SD (*n* = 3, hCol; *n* = 3, control). *P < 0.05; **P < 0.01 versus control at each time point.

Collagen, as a bioactive component *in vitro*, has been explored in wound care supplements for post-treatments to provide a microenvironment for cell growth and promote matrix remodeling [44]. Hereby, a full-thickness wound area was created on tail skin per mouse (post-operative day 0) and wound closure was measured by the area of the wound bed over time, normalized to the initial wound after treatment (Figure 6A). As seen in the photos of wound areas, the mice of the hCol group healed more quickly as compared to the commercial control. Specifically, the wound closure values for hCol (*n* = 3) versus control (*n* = 3) mice were 79% versus 3% at day 7(*P* < 0.05), 76% versus 56% at day 14 (*P* < 0.05) and 55% versus 26% at day 21 (*P* < 0.01) in Figure 6B. For the late stage of wound healing, hCol showed epidermis regeneration and healthy dermis with no inflammation, while the control group displayed inflammation in the regenerated epidermis at day 14. At day 21, hCol displayed a uniform thickness of the newly generated epidermis while the control group did not (Figure 6C).

## Discussion

Each collagen α chain consists of three domains: N-propeptide, uninterrupted triple helix and C-telopeptide [45, 46]. The specific glycosylated hydroxylysine residues (O-linked glycosylation) are formed in the triple helix region of proα chains [47]. Each chain of C-propeptide is glycosylated by high-mannose asparagine-linked oligosaccharide (N-glycosylation) and this domain is further stabilized by interchain-disulfide bonds [48]. After these and other modifications, two proα1 chains and one proα2 chain are folded into a triple helix structure from the C- to the N-terminus [49]. Subsequent to folding, an offset, parallel packing of triple-helices is generated to form microfibrils [50]. The three-dimensional structure is featured by a 67-nm D-periodic repeat [51]. The hierarchical packing structure is maintained *in vivo* through the attachment of stabilizing ligands to both the fibril and fibril bundle (s) [52]. Due to its bioactive domains and unique hierarchical structures at different length scales, collagen plays a dominant role in regulating cell function and directing tissue development [43, 53]. Therefore, the components and multi-scale structures of hCol were verified by SDS-PAGE, HPLC-MS, FTIR, Raman, CD spectroscopy, SEM and TEM in this work to confirm its native-like properties.

To collect hCol, cells and PES materials were removed and from SEM images, the microstructure of ECM was still complete after the treatment. The SDS-PAGE analysis showed high purity of type I collagen in hCol. HPLC-MS further demonstrated glycosylation and disulfide linkages occurred at the same sites on collagen alpha chains in hCol as compared to native bCol I. This implied hCol had correct post-translational modifications and folding of domains prior to triple helix formation as compared to native bCol I. Although the N-glycan structure was different between hCol and bCol I, it could result from different features of N-glycosylation in the two groups [54, 55]. The cell culture process could also influence N-glycosylation structure of proteins derived from cells [56, 57]. In the future, cell culture process should be well controlled regarding to N-gycosylation structures in human native collagen. For collagen assembling into a triple helix structure, FTIR spectra displayed a lower degree of triple helix in hCol, which was in parallel to the CD spectroscopy results where the thermal stability of triple helix structure in hCol was slightly lower than bCol I. This may imply a slightly different peptide sequence, length and modifications in collagen for hCol I and bCol. Nevertheless, the degradation temperature of the triple helix in hCol was above normal physiological temperature, indicating hCol had the capability to maintain its function in human body. More importantly, Raman spectra showed the predominance of triple helix structure in hCol along with other secondary structures such as alpha helix and beta sheet. The globular heads in the C1 domain of the collagen chain primarily contain beta-sheet structure and type III collagen has a lower alpha helix and triple helix than type I [58]. Hence, it could result in a smaller percentage of alpha and triple helix in hCol with a certain amount of type III collagen as seen in Figure 3C and D. For other regions, although the Raman positions of peaks in C–C, C–S stretching region, amide III and C–H bending position were different to some degree between bCol I and hCol, it could attributed to spectral variability and broad spectral features in biological samples [59, 60]. For fibrillar structure, SEM and TEM showed a 68-nm D-periodicity of collagen fibril in hCol. This banded structure results from the axial stagger of adjacent tropocollagen molecules, recorded as gap and overlap regions. The regular array of gap and overlap regions indicated a regular assembly of collagen monomers formed in hCol. Overall, type I collagen was predominant in hCol with a few amount of type III collagen, which was similar to the composition of the human body with less than 20% of type III collagen.

After confirming native-like structural features in hCol, the effects of hCol on cell behavior and phenotype were examined. As one of the ideal candidates for use in regenerative medicine protocols, hASCs have the capability to differentiate into particular lineage cells that replace damaged cells and contribute to tissue regeneration [61]. However, the successful use of MSC in therapy requires developing well-defined methods for cell growth and directing differentiation [62]. These results showed that hCol promoted hASC viability, proliferation and migration. The migration ability was greater for hCol than bCol I, which could result from a higher content of integrin recognition sequences in hCol than bCol I, leading to a higher probability of interacting with hASC integrins. Meanwhile, collagen triple helices as guidance structures for contacting cells, and the degradation of triple helix structure resulted in reduced migration ability [63]. In this work, heating, hydrogen peroxide and radiation treatment can cleave the hydrogen and covalent bonds to destabilize the triple helix of hCol, which suppresses the cell migration ability. However, alkaline treatment had less effect on the triple helix structure of collagen as previously reported in several literatures [64, 65]. The alkaline treatment can remove the telopeptides of the collagen molecules and break off additional crosslinking in the triple helical regions without damaging the integrity of the triple helix, which can retain the integrin recognition sites for binding cells without much impact on cell migration as compared to no treatment. Besides, hCol promoted adipocyte differentiation as compared to hASCs cultured in AIM by a higher number of mature adipocytes through enhanced expression of LPL and PPARγ2 genes that play an important role in adipocyte differentiation. Moreover, hCol can accelerate cutaneous wound healing as compared to commercial recombinant collagen products by causing less inflammation as seen in the HE staining images. This could be due to the more native-like property of hCol compared to recombinant collagen, as hCol was directly derived by cells *in vitro* that contained all necessary domains for bioactivity. Whether the healing capability is related to the native triple helix structure of hCol should be investigated in the future. Meanwhile, several studies have demonstrated that type I collagen promoted cell migration, cell attachment and MSC adipogenesis by more cell-to-cell surface contact points and interaction with cellular receptors (e.g. α2β1, DDR2, etc.) [1, 17]. All of these suggest that a platform to produce native-like collagen from cell-derived matrix was provided and hCol could be potentially used as *in vitro* cell culture model in tissue engineering or cosmetics [66]. In the future, the mechanism of how collagen structure promotes cell phenotype and skin repair needs to be investigated in combination with more molecular biology tests.

## Conclusion

The biochemical characterization in this study verified a high purity of type I collagen in hCol. The structural examinations at different length scales confirmed native-like hierarchical structures in hCol, consisting of triple helix structures with the native form of post-translational modification, assembled into supramolecular fibrils with banded 68 nm repeating structures. The *in vitro* cell culture with hCol indicated its noticeable influence on hASC behaviors and phenotype, including promoting cell proliferation, migration and hASC adipogenesis. Besides, hCol also accelerated cutaneous wound closure compared to available commercial collagen products, implying that hCol can serve as a prospective candidate for tissue repair as native collagen. This study can provide a strategy to harvest native-like collagen and give detailed evidence to fill in the knowledge gap of structure and its effect on cell behaviors, which might support its application in tissue repair.

## Supplementary Material

rbae008_Supplementary_Data
