# Supplementary material for: Human extracellular matrix (ECM)-like collagen and its bioactivity
Source: Regen Biomater. 2024 Feb 1;11:rbae008. doi: 10.1093/rb/rbae008 (PMC10965421; doi:10.1093/rb/rbae008)
Supplement: rbae008_Supplementary_Data [file rbae008_supplementary_data.pdf]

Supplementary materials

Human Extracellular Matrix (ECM)-like Collagen and its Bioactivity

Hui Zhou[1], Wenwei Li[2], Lixin Pan[2], Tianci Zhu[2], Teng Zhou[2], E Xiao[2*] and Qiang Wei[1,2*]

1. State Key Laboratory of Polymer Materials and Engineering, College of Polymer Science and Engineering, Sichuan University, Chengdu 610065, China

2. Hunan Maybio Bio-Pharmaceutical Co., Ltd., Changsha 410000, China

[*]Address correspondence to:wei@scu.edu.cn(Q.W.); xiaoe1986@vip.163.com(E.X.)

For Review Only

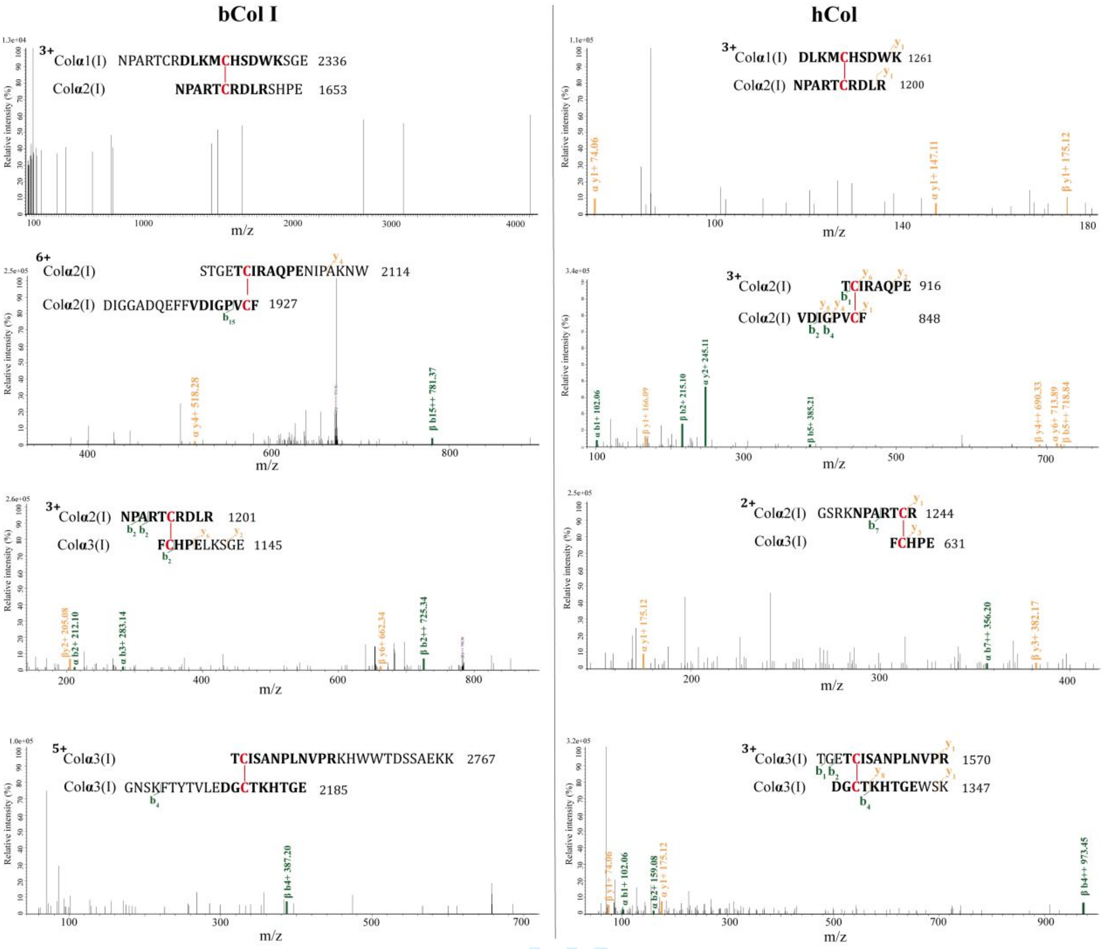

Fig S1. High similarity in sequencing positioning of disulfide linkages between bCol I and hCol. HCD spectra of different charge disulfide-linked peptide ions for bCol I and hCol resulting between collagen α1 and α2 chain(first row), α2 chains(second row), α2 to α3 chain(third row) and α3 chains(last row). The annotation in green and orange color denote fragments from the cleavage of amide bonds that retain N-terminus and C-terminus respectively.

Table S1. The major peak assignment in FTIR spectra of bCol I and hCol

Region	Peak wavenumber (cm^{-1})		Assignment
	bCol I	hCol	
Amide A	3313	3313	υ(C=O), υ(N-H)
Amide B	3091	3086	υ(N-H)
Amide I	1650	1650	υ(C=O)
Amide II	1549	1545	δ(N-H), υ(C-N)
Amide III	1239	1238	δ(N-H), υ(C-N)

Table S2. Raman characteristic band assignment in bCol I and hCol

Region	Peak wavenumber (cm^{-1})		Assignment
	bCol I	hCol	
υ(C-S), υ(C-C) & υ(S-S)	515, 564	520, 564	υ(S-S) of collagen
	624, 659	613, 657	τ(C-C) of cystine, tyrosine, phenylalanine (type I collagen)
	690, 735	690,737	υ(C-S) of proteins
	753	757	δ(ring) of tryptophan
	806, 820	796, 816	υ(C-C) of proline, tyrosine, hydroxyproline (collagen)
	863, 881	855, 887	υ(C-C) of proline, tyrosine and δ(ring) of tryptophan
	932, 946, 964, 980	924, 940, 972	υ(C-C) of proline, valine and protein backbone
	1002	1007	υ(C-C) of phenylalanine
	1075	1070, 1079	υ(C-C) of skeletal backbone in lipid
Amide III	1236, 1263, 1287	1237, 1245 1273	δ(N-H), υ(C-N) of glycine backbone & proline side chains
CH bending	1310, 1330	1300, 1328	τ(CH_2), τ(CH_3), ω(CH_2CH_3) of collagen
	1451	1452	δ(CH_2), τ(CH_2CH_3) of proteins
Amide I	1668, 1696	1667, 1688	υ(C=O) amide I of collagen

For Review Only
